# Tips for efficiently maintaining pET expression plasmids

**DOI:** 10.1007/s00294-023-01276-0

**Published:** 2023-11-08

**Authors:** Diana Khananisho, Alister J. Cumming, Daria Kulakova, Patrick J. Shilling, Daniel O. Daley

**Affiliations:** Department of Biochemistry and Biophysics, Stockholm University, Stockholm, Sweden

**Keywords:** pET expression plasmid, Tn903, Aminoglycoside-3’-phosphotransferase, Tn3, ß-Lactamase, Bacterial cell factory, Plasmid maintenance, Plasmid stability, Plasmid instability

## Abstract

pET expression plasmids are widely used for producing recombinant proteins in *Escherichia coli.* Selection and maintenance of cells harboring a pET plasmid are possible using either a Tn3.1-type genetic fragment (which encodes a ß-lactamase and confers resistance to ß-lactam antibiotics) or a Tn903.1-type genetic fragment (which encodes an aminoglycoside-3’-phosphotransferase and confers resistance aminoglycoside antibiotics). Herein we have investigated how efficiently pET plasmids are maintained using these two fragments. The study reveals that pET plasmids are efficiently maintained with both Tn3.1 and Tn903.1 genetic fragments prior to the induction of recombinant protein production, and over short induction times (i.e., 2 h). However, over longer induction times (i.e., 20 h), the efficiency of plasmid maintenance depends on the host strain used, and the type of antibiotic selection cassette used. Based on our collective observations, we have 2 general tips for efficiently maintaining pET plasmids during recombinant production experiments.

- Tip #1: Use a strain with lowered levels of the T7 RNA polymerase, such as C41(*DE3*). pET plasmids will be efficiently maintained over long induction times with both the Tn3.1 and Tn903.1 genetic fragments, regardless of whether antibiotics are present during cultivation.
- Tip #2: If a strain with higher levels of T7 RNA polymerase strain is necessary, such as BL21(*DE3*)), keep induction times short or use a plasmid containing a Tn903.1-type fragment and select with kanamycin.

**Supplementary Information:**

The online version contains supplementary material available at 10.1007/s00294-023-01276-0.

## Introduction

pET plasmids are popular in both academia and industry for recombinant protein production (Heyde and Nørholm 2021; Rosano et al. 2019; Rosano and Ceccarelli 2014; Shilling et al. 2020). They contain φ10 promoter (T7p) and terminator (T7t), which are recognized by a phage-derived T7 RNA polymerase in the host strain. Recombinant coding sequences cloned downstream of T7p are therefore transcribed by the T7 RNA polymerase and translated by the host cell ribosomes. As the T7 RNA polymerase is orthogonal and approximately five times faster than the *E. coli* RNA polymerase (Chamberlin and Ring 1973; Golomb and Chamberlin 1974), large amounts of recombinant RNA are generated in a short period of time (Studier et al. 1990), and the recombinant protein can constitute up to 50% of the cells protein content (Mierendorf et al. 1998).

The first-generation pET plasmids (pET1–pET5) were developed using a pBR322 backbone, which contained a Tn3.1 fragment harboring the coding sequence for ß-lactamase (*bla*) (Rosenberg et al. 1987). These plasmids could therefore be selected and maintained using ampicillin and other ß-lactam antibiotics (Bolivar et al. 1977; Sutcliffe 1979). In the later generation pET9 expression plasmid, the Tn3.1 fragment was replaced by a Tn903.1 transposon from *Streptomyces kanamyceticus*, which contains the coding sequence for the aminoglycoside-3’-phosphotransferase type Ia enzyme (*aph*) (Oka et al. 1981; Studier et al. 1990; Umezawa 1979). Selection and maintenance of cells harboring this plasmid were therefore possible using kanamycin or other aminoglycoside antibiotics. When the early-generation pET plasmids were subsequently expanded by commercial vendors, Tn3.1 and Tn903.1 fragments acquired some nucleotide mutations, but the basic architecture remained essentially unchanged. Currently, there are 55 pET expression plasmids containing a Tn3.1-based fragment (Cumming et al. 2022), and 21 containing a Tn903.1-based fragment (Table 1).

**Table 1:** Antibiotic resistance fragments used in commercially available pET expression plasmids

Fragment	Resistance	Seq^A^	Expression plasmids using the fragment^I^
Tn3.1	ß-lactams	B	pET1, pET2, pET3, pET4, pET5, pET6, pET7, pET8, pET11a-d, pET14b, pET15b, pET16b, pET17b, pET19b, pET-DEST42, pET100/D-TOPO, pET100/D-LacZ, pET101/D-LacZ, pET101/D-TOPO, pET102/D-LacZ, pET102/D-TOPO, pET104-DEST, pET104/GW/LacZ, pET104.1-DEST, pET104.1/D/GW-LacZ, pET151/D-TOPO, pET151/D/LacZ, pET160-DEST, pET160/GW/D-TOPO, pET161/GW-CAT, pET300/NT-GW/Ras Kinase, pET300/NT-DEST, pET301/CT-DEST, pET302/NT-his
Tn3.3	ß-lactams	C	pET20b( +), pET21a-d( +), pET22( +), pET23a-d( +), pET25b( +), pET31b( +), pET32a-c( +), pET32 Ek/LIC, pET32 Xa/LIC
Tn3.7	ß-lactams	D	pETduet-1, pET43 Ek/LIC, pET43.1a( +), pET44a-c( +), pET45b( +), pET46 Ek/LIC, pET51b( +), pET51 Ek/LIC, pET52( +), pET52 Ek_LIC
Tn3.14	ß-lactams	E	pET303-CT-His-Rac Kinase, pET303-CT-His
Tn903.1	Aminoglycosides	F	*pET9a,b,c,d*
Tn903.2	Aminoglycosides	G	*pET24a,b,c pET26b, pET27b, pET28a,b,c pET29a,b,c pET30a, pET33b, pET39b, pET40b, pET41a,b,c pET41 Ek/LIC, pET42a,b,c, pET47b, pET49b, pET-SUMOpro Kan, pET-SUMOpro3 Kan, pET-SUMOstar Kan, pET-SUMO, pET-SUMO/CAT*
Tn903.3	Aminoglycosides	H	*pET50b*

^A^Nucleotide sequence of the Tn3.1 fragment is shown in “B” with the *bla* coding sequence highlighted. It was described in (Rosenberg et al. 1987) and obtained from Addgene. Nucleotide changes from this fragment are indicated by the letter’s “C” to “E”. Nucleotide sequence of the Tn903.1 fragment was obtained as an 867 bp fragment from *pUC4KISS* (Studier and Moffatt 1986). It consists of 50 bp upstream of the *aph* initiation codon, the *aph* gene (816 bp) and 1 bp after the termination codon. The full sequence is shown in “F”, with the *aph* gene highlighted. Nucleotide changes from this fragment are indicated by the letter’s “G” and “H”

^B^5’TTCTTGAAGACGAAAGGGCCTCGTGATACGCCTATTTTTATAGGTTAATGTCATGATAATAATGGTTTCTTAGACGTCAGGTGGCACTTTTCGGGGAAATGTGCGCGGAACCCCTATTTGTTTATTTTTCTAAATACATTCAAATATGTATCCGCTCATGAGACAATAACCCTGATAAATGCTTCAATAATATTGAAAAAGGAAGAGT**ATGAGTATTCAACATTTCCGTGTCGCCCTTATTCCCTTTTTTGCGGCATTTTGCCTTCCTGTTTTTGCTCACCCAGAAACGCTGGTGAAAGTAAAAGATGCTGAAGATCAGTTGGGTGCACGAGTGGGTTACATCGAACTGGATCTCAACAGCGGTAAGATCCTTGAGAGTTTTCGCCCCGAAGAACGTTTTCCAATGATGAGCACTTTTAAAGTTCTGCTATGTGGCGCGGTATTATCCCGTGTTGACGCCGGGCAAGAGCAACTCGGTCGCCGCATACACTATTCTCAGAATGACTTGGTTGAGTACTCACCAGTCACAGAAAAGCATCTTACGGATGGCATGACAGTAAGAGAATTATGCAGTGCTGCCATAACCATGAGTGATAACACTGCGGCCAACTTACTTCTGACAACGATCGGAGGACCGAAGGAGCTAACCGCTTTTTTGCACAACATGGGGGATCATGTAACTCGCCTTGATCGTTGGGAACCGGAGCTGAATGAAGCCATACCAAACGACGAGCGTGACACCACGATGCCTGCAGCAATGGCAACAACGTTGCGCAAACTATTAACTGGCGAACTACTTACTCTAGCTTCCCGGCAACAATTAATAGACTGGATGGAGGCGGATAAAGTTGCAGGACCACTTCTGCGCTCGGCCCTTCCGGCTGGCTGGTTTATTGCTGATAAATCTGGAGCCGGTGAGCGTGGGTCTCGCGGTATCATTGCAGCACTGGGGCCAGATGGTAAGCCCTCCCGTATCGTAGTTATCTACACGACGGGGAGTCAGGCAACTATGGATGAACGAAATAGACAGATCGCTGAGATAGGTGCCTCACTGATTAAGCATTGGTAA** CTGTCAGACCAAGTTTACTCATATATACTTTAGATTGATTTAAAACTTCATTTTTAATTTAAAAGGATCTAGGTGAAGATCCTTTTTGATAATCTCATGACCAAAATCCCTTAACGTGAGTTTTCGTTCCACTGAGCGTCAGACCCC-3’

^C^As per nucleotide sequence in “B” except G244 to A mutation in *bla* (V82 to I); nucleotides 1-76 missing from 5’UTR

^D^As per nucleotide sequence in “B” except G244 to A mutation in *bla* (V82 to I); 545 to T mutation in *bla* (A182 to V); nucleotide mutation in 5’UTR (-20 A to C); nucleotide mutation in 5’UTR (-93 A to C); nucleotides 1-110 missing from 5’UTR; nucleotides 1172-1216 missing from 3’UTR

^E^As per nucleotide sequence in “B” except G244 to A mutation in *bla* (V82 to I); nucleotides 1-86 missing from 5’UTR

^F^5’CATGAACAATAAAACTGTCTGCTTACATAAACAGTAATACAAGGGGTGTTATGAGCCATATTCAACGGGAAACGTCTTGCTCGAGGCCGCGATTAAATTCCAACATGGATGCTGATTTATATGGGTATAAATGGGCTCGCGATAATGTCGGGCAATCAGGTGCGACAATCTATCGATTGTATGGGAAGCCCGATGCGCCAGAGTTGTTTCTGAAACATGGCAAAGGTAGCGTTGCCAATGATGTTACAGATGAGATGGTCAGACTAAACTGGCTGACGGAATTTATGCCTCTTCCGACCATCAAGCATTTTATCCGTACTCCTGATGATGCATGGTTACTCACCACTGCGATCCCCGGGAAAACAGCATTCCAGGTATTAGAAGAATATCCTGATTCAGGTGAAAATATTGTTGATGCGCTGGCAGTGTTCCTGCGCCGGTTGCATTCGATTCCTGTTTGTAATTGTCCTTTTAACAGCGATCGCGTATTTCGTCTCGCTCAGGCGCAATCACGAATGAATAACGGTTTGGTTGATGCGAGTGATTTTGATGACGAGCGTAATGGCTGGCCTGTTGAACAAGTCTGGAAAGAAATGCATAAGCTTTTGCCATTCTCACCGGATTCAGTCGTCACTCATGGTGATTTCTCACTTGATAACCTTATTTTTGACGAGGGGAAATTAATAGGTTGTATTGATGTTGGACGAGTCGGAATCGCAGACCGATACCAGGATCTTGCCATCCTATGGAACTGCCTCGGTGAGTTTTCTCCTTCATTACAGAAACGGCTTTTTCAAAAATATGGTATTGATAATCCTGATATGAATAAATTGCAGTTTCATTTGATGCTCGATGAGTTTTTCTAAG ’3

^G^As per nucleotide sequence in “F” except silent nucleotide mutation in position 85 G-> T; silent nucleotide mutation in position 602 G-> A

^H^As per nucleotide sequence in “F” except silent nucleotide mutation in position 85 G-> T; silent nucleotide mutation in position 602 G-> A; silent nucleotide mutation in position 359 G-> C; silent nucleotide mutation in position 479 C-> T

^I^Nucleotides sequences were not found for pET1, pET2, pET3, pET4, pET5, pET6, pET7, pET8 so they were assumed to be the same as pBR322 and pET11a-d

In this study, we have investigated whether pET expression plasmids using the Tn3.1 and Tn903.1 fragments are efficiently maintained in cells, or whether the cultures become over-run with plasmid-less cells when these fragments are used. Previous work has indicated that they are maintained well in some experiments but not in others (Baheri et al. 2001; Cumming et al. 2022; Dumon-Seignovert et al. 2004; Pan and Malcolm 2000; Sieben et al. 2016). However, a clear picture is conspicuously absent from the literature as the aforementioned studies have not addressed the question in a broad sense. Herein we have therefore looked at the efficiency of plasmid maintenance for two of the most commonly used pET expression plasmids (pET28a, pET15b), using both the Tn3.1-type and the Tn903.1-type fragments, in two of the most commonly used strains (BL21(*DE3*), C41(*DE3*)). We have done this both with and without antibiotic selection, using two common induction conditions (0.5 mM IPTG for 2 or 20 h).

## Materials and methods

### Database searching

Nucleotide sequences of Tn903.1 and Tn3.1 fragments in commercially available pET expression plasmids were obtained from Addgene (https://www.addgene.org) and are shown in Table 1. Alignments were performed using the nucleotide BLAST (nBLAST) service from the National Center for Biotechnology Information (https://blast.ncbi.nlm.nih.gov/Blast.cgi), using the Tn903.1 fragment from the pET9 plasmid (Studier et al. 1990) and Tn3.1 fragment from pET3 (Rosenberg et al. 1987) as the reference.

### Molecular cloning

Construction of pET28a-sfGFP (Tn903.1), pET28a-Mth1 (Tn903.1), pET15b-sfGFP (Tn3.1), and pET15b-Mth1 (Tn3.1) was described in (Shilling et al. 2020). Construction of the pET28a-sfGFP (Tn3.1) as well as pET15b-sfGFP (Tn903.1*)* was done by (1) PCR amplification of the pET15b-sfGFP and pET28a-sfGFP backbones using forward and reverse primers annealing on either side of the antibiotic resistance fragments in order to create a plasmid backbone absent of the cassette. The PCR cycle used was composed of 30 cycles of 95 °C for 30 s, 49 °C for 30 s, 72 °C for 360 s. (2) The Tn903.1 and Tn3.1 fragments were PCR-amplified from pET28a-sfGFP (Tn903.1) and pET15b-sfGFP (Tn3.1) using forward and reverse primers annealing on either side of the fragment and containing an overhang region. The PCR cycle was composed of 30 cycles of 95 °C for 30 s, 46 °C for 30 s, 72 °C for 120 s. The subsequent PCR product was purified via *in-gel* extraction and introduced to MC1061 cells for in vivo assembly (Watson and García-Nafría, 2019).

All polymerase chain reactions were carried out with Q5 DNA polymerase (New England Biolabs, USA). All primers are described in (Supplementary information, Table S1). DNA sequencing and oligonucleotide synthesis were performed by Eurofins Scientific (Eurofins Genomics, Germany).

### Plasmid selection and maintenance

To assess transformation efficiency, 50 ng of each plasmid was transformed into a 50 μL aliquot of chemically competent BL21(*DE3*) cells (B F^−^ *ompT gal dcm lon hsdS*_*B*_ (*r*_*B*_^*−*^*m*_*B*_^*−*^) *λ*(DE3 [*lacI lac UV5-T7p07 ind1 sam7 nin5*]) using a standard heat shock protocol. An aliquot of the cells was plated on LB agar, the appropriate antibiotic at the appropriate concentration (Tn903.1 at 50 μg/mL kanamycin, Tn3.1 at 100 μg/mL ampicillin). Images were taken using the upper white light in a GenoPlex (VWR International), and the number of colonies was counted using the OpenCFU software (Geissmann 2013).

To assess plasmid maintenance, a single colony of BL21(*DE3*), or the derivative strain C41(*DE3*) (Miroux and Walker 1996), containing a pET expression plasmid was grown in 5 mL of LB media for 16–20 h at 37 °C with agitation at 185 rpm in a 24-well plate. The cultures were back-diluted 1:100 in 5 mL LB media and grown to an OD_600_ of approximately 0.5. In certain experiments, antibiotics were omitted, otherwise the appropriate antibiotic was included at the appropriate concentration (see above). The cells were either induced with 0.5 mM IPTG, or simply cultured, then plated out on LB agar with the appropriate antibiotic at the appropriate concentration at designated times points. Images were taken using the upper white light in a GenoPlex (VWR International), and the number of colonies  were counted using the OpenCFU software (Geissmann 2013).

### Minimum inhibitory concentration

A single colony of BL21(*DE3*) was inoculated into 5 ml of LB media with 50 μg/mL of kanamycin (Tn903.1) and incubated overnight at 37 °C with agitation at 185 rpm. The cultures were back-diluted 1:100 the following morning in fresh LB media with kanamycin in a 5 mL 24-well plate and grown to an OD_600_ between 0.3 and 0.6. The cultures were then serially diluted and 100 μL was plated onto LB agar plates with varied concentrations of kanamycin. Images were taken using the upper white light in a GenoPlex (VWR International), and the number of colonies were counted using the OpenCFU software (Geissmann 2013).

### Protein expression

A single colony of BL21(*DE3*) containing a pET expression plasmid was grown in 5 mL of LB media with the appropriate antibiotic at the appropriate concentration (see sub-section entitled ‘Plasmid selection and maintenance*’*) for 16–20 h at 37 °C with agitation at 185 rpm in a 5 mL 24-well plate. On the following day, the cultures were back-diluted 1:100 in 5 mL LB media with the same concentrations of antibiotic in a 5 mL 24-well plate. Growth was followed by taking 200 μL of cell culture into a 96-well plate (Thermo Scientific) and measuring absorbance at 600 nm (OD_600_) in a Spectramax M2e (Molecular Devices). Cells were induced with 0.5 mM IPTG at an OD_600_ between 0.3 and 0.6 and incubated for an additional 20 h at 37 °C with agitation at 185 rpm.

### sfGFP fluorescence measurements

Fluorescence measurements were carried out as described previously (Daley et al. 2005). In short, 1 mL of the culture was pelleted at 13,000 × *g* for 1 min, and the cells were re-suspended in 200 μL of buffer (50 mM Tris–HCl, pH 8, 200 mM NaCl, 15 mM EDTA) and incubated at room temperature for 2 h. The cell suspension was then transferred to a 96-well optical bottom black-wall plate (Thermo Scientific), and the fluorescence was measured at excitation/emission wavelengths of 485/513 nm in a SpectraMax Gemini (Molecular Devices). Fluorescence was normalized against OD_600_.

### SDS-PAGE and Western blotting

SDS-PAGE was performed with a Tris–glycine 12% acrylamide gel, cast to a thickness of 1 mm and run using the Hoefer SE160 Mighty Small II Deluxe Mini Vertical Protein Electrophoresis Unit at 100 V for 3 h. For Western blotting, proteins were transferred into a nitrocellulose membrane using the semi-dry Trans-Blot SD cell (Bio-Rad) at 15 V for 1 h. The membrane was incubated in 5% (w/v) non-fat milk (PanReac AppliChem) in Tris-buffered saline (TBS) (50 mM Tris, pH 7.4, 200 mM NaCl) for either 1 h or overnight. Recombinant proteins bearing a His-tag were probed using a 1: 1000 diluted HisProbe–HRP conjugate (15,165, Thermo Scientific), and the SuperSignal West Pico PLUS chemiluminescence substrate was used as a substrate. Images were visualized on an Azure c600 Imaging System (Azure Biosystems).

### Kanamycin activity assay

BL21(*DE3*) cells harboring pET15-sfGFP (Tn3.1) or pET28a-sfGFP (Tn903.1) were inoculated into LB media supplemented with the appropriate antibiotic at the appropriate concentration (see sub-section entitled ‘Plasmid selection and maintenance*’*) for 16–20 h at 37 °C with agitation at 185 rpm. To collect the spent media, a 5 mL aliquot of BL21(*DE3*) harboring pET28a-sfGFP (Tn903.1) was pelleted by centrifugation at 4 000 x*g* for 10 min, then sterile-filtered through a 0.2 µm filter (Whatman) and supplemented with 10% (w/v) glucose. BL21(*DE3*) harboring pET15b-sfGFP (Tn3.1) were diluted 1:100 into the spent media that had been supplemented with 100 µg/mL ampicillin. Separately, cultures of BL21(*DE3*) harboring pET15b-sfGFP were also inoculated 1:100 into fresh LB media with 100 µg/mL ampicillin. Growth was monitored by measuring the OD_600_ each hour for five hours in a 96-well SpectraMax *m2e* plate reader (Molecular Devices).

## Results

### Maintenance of pET plasmids in BL21(DE3)

**Fig. 1:**
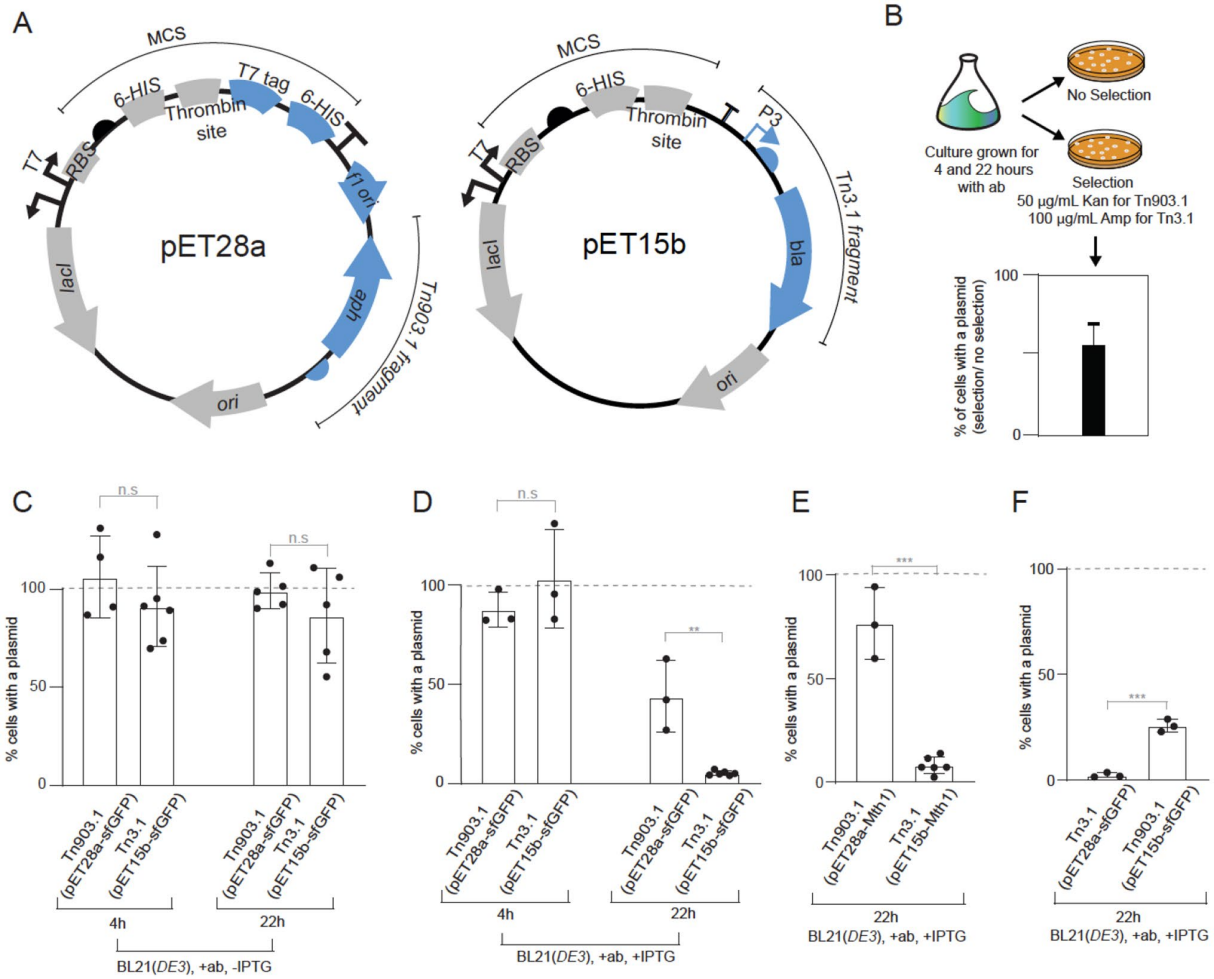
Maintenance of pET plasmids in BL21(*DE3*) harboring Tn903.1 and Tn3.1 (cultivated in LB media supplemented with antibiotics). **A** Schematic representation of the pET28a and pET15b expression plasmids, which harbor the Tn903.1 (*aph*, KanR) or Tn3.1 (*bla*, AmpR) fragments respectively. Salient features are marked. Regions marked in gray are identical in nucleotide sequence between both plasmids. Regions marked in blue are unique to the expression plasmid. **B** Schematic representation of the experimental workflow (adapted from (Cumming et al. 2022). Plasmid maintenance was determined from liquid cultures supplemented with antibiotics, then plated on LB agar plates with and without antibiotic selection. Plasmid maintenance = [# colonies on plate with antibiotic / # colonies on plate without antibiotic] × 100. **C** Plasmid maintenance for BL21(*DE3*) harboring either pET28a-sfGFP (Tn903.1) or pET15b-sfGFP (Tn3.1) in the absence of induction with IPTG. **D** As for panel (C) except that cells were induced with 0.5 mM IPTG. **E** As for panel (D) except that BL21(*DE3*) were harboring pET28a-Mth1 (Tn903.1) or pET15b-Mth1 (Tn3.1). Mth1 is a putative human cancer target that sanitizes the oxidized dNTP pools (Gad et al. 2014b).** F** As for panel (D) except the resistance cassettes were swapped between the plasmids: pET28a-sfGFP (Tn3.1) or pET15b-sfGFP (Tn903.1). A statistically significant difference of *p* < 0.05, *p* < 0.005 or *p* < 0.0005 (two-tailed Student’s t-test) is denoted by *, ** and *** respectively. n.s means that the difference was not statistically significant. Note that similar experiments with Tn3.1 in panels C, D and E were presented in (Cumming et al. 2022). They were repeated here for reference

pET28a and pET15b are two of the most popular pET expression plasmids (Shilling et al. 2020). pET28a contains a Tn903.1 fragment that enables selection and maintenance of cells carrying the plasmid using kanamycin (Fig. 1A, left panel). pET15b is similar, except that it lacks a C-terminal T7-His6 tag and the F1 *ori* and contains a Tn3.1 fragment that confers resistance to ß-lactam antibiotics (Fig. 1A, right panel). Initially, we compared the transformation efficiency of pET28a-sfGFP (Tn903.1) and pET15b-sfGFP (Tn3.1) by transforming 50 ng of each plasmid into the BL21(*DE3*) strain and plating on the appropriate antibiotic. Colony counting indicated that plasmid transformation worked equally when both expression plasmids were used (Supplementary information Figure S1). To assess how efficiently cells maintained the two expression plasmids, colonies were inoculated into LB media (with the appropriate antibiotic) and cultivated at 37 °C. Aliquots were taken after 4 and 22 h of cultivation and plated on LB agar. This enabled an assessment of the proportion of viable cells that were resistant to the antibiotic (hereafter referred to as plasmid maintenance; Fig. 1B). BL21(*DE3*) maintained both plasmids with approximately 100% efficiency after 22 h of cultivation in the presence of antibiotics in the culture media (Fig. 1C).

When the experiment was repeated and recombinant sfGFP production was induced at mid exponential phase (2 h after back-dilution) with 0.5 mM IPTG for 2 h, we observed that approximately 100% of the cells still maintained both of the plasmids. However, after 20 h of induction (i.e., 22 h of cultivation), approximately 50% of BL21(*DE3*) cells had maintained pET28a-sfGFP (Tn903.1) and < 15% had maintained pET15b-sfGFP (Tn3.1) (Fig. 1D) (see also (Cumming et al. 2022)). A difference in the efficiency of plasmid maintenance was also observed when the coding sequence for the human cancer target Mth1 (Gad et al. 2014) was expressed from pET28a-Mth1 (Tn903.1) compared to pET15b-Mth1 (Tn3.1) for 20 h (Fig. 1E).

To eliminate the possibility that the differences in plasmid maintenance were caused by the pET15b and pET28a backbones rather than by the genetic fragments used for selection and maintenance, the Tn903.1 and Tn3.1 fragments were swapped, and plasmid maintenance was again assessed during production of recombinant sfGFP. Over a 20-h induction period (22 h of cultivation), approximately 2% of BL21(*DE3*) cells had maintained pET28a-sfGFP (Tn3.1) and 25% had maintained pET15b-sfGFP (Tn903.1) (Fig. 1F). The data presented indicate that during recombinant protein production experiments, pET plasmids are not efficiently maintained in BL21(*DE3*) cells. However, plasmids containing the Tn903.1 fragment (and selected on kanamycin) were maintained more efficiently in BL21(*DE3*) than those containing Tn3.1 (and selected on ampicillin).

Increased plasmid maintenance did not lead to increased protein production titers. When sfGFP and Mth1 yields were quantified by in cell fluorescence and Western blotting respectively, there were no major differences in protein yields between BL21(*DE3*) cells harboring pET expression plasmids with either the Tn903.1 fragment or the Tn3.1 fragment (Supplementary information Figure S2). This observation is most likely explained by the finding that, over long induction times, BL21(*DE3*) cells that maintain pET expression plasmids either down-regulate or shut-off the pET system via mutations because it is toxic to the cell (James et al. 2021; Kwon et al. 2015a; Schlegel et al. 2015).

Why are pET plasmids containing Tn903.1 (and selected on kanamycin) maintained more efficiently in BL21(*DE3*) during recombinant protein production experiments than those containing Tn3.1 (and selected on ampicillin)? We reason that this difference can be attributed to the half-life (*t*_*1/2*_) of the respective antibiotics. Previous work has demonstrated that ampicillin in the culture media is rapidly degraded by ß-lactamase (*t*_*1/2*_ approx. 6 min following back-dilution) (Cumming et al. 2022)). Cells that lose a pET plasmid containing the Tn3.1 fragment are therefore not selected against after approx. 70 min (following back-dilution)(Cumming et al. 2022). These cells can subsequently dominate the culture because they are freed from the metabolic burden of plasmid maintenance and recombinant protein production, which gives them a growth advantage. In contrast, kanamycin is active in spent media after + 20 h of culturing (i.e., it can select against cells without a plasmid) (Supplementary information Figure S3). Cells that lose a pET plasmid containing the Tn903.1 fragment are therefore selected against and cannot dominate the culture, even though they are freed from the metabolic burden of recombinant protein production and have a growth advantage. It is worth noting however, that approx. 50–75% of cells in the culture did not survive when re-plated on 50 ug /mL kanamycin. Possible reasons for this are discussed below.

The importance of antibiotic selection for plasmid maintenance is well established. However, many pET users do not have the luxury of using antibiotics during production experiments (i.e., in industrial settings where antibiotic residues and metabolites are unwanted). We repeated key experiments in the absence of antibiotics and observed that pET28a-sfGFP (Tn903.1) and pET15b-sfGFP (Tn3.1) were maintained with approximately 100% efficiency in BL21(*DE3*) over a 20-h period. However, when recombinant protein production was induced for 20 h (22 h of culturing), < 10% of the cells in the cultures maintained the plasmid (Supplementary information Figure S4). This observation further emphasizes the importance of selection during recombinant protein production.

In summary, these data indicate that pET plasmids harboring both Tn903.1 and Tn3.1 fragments are efficiently maintained over short induction times in BL21(*DE3*). Over longer induction times, the cultures become overgrown with cells lacking plasmid. Nevertheless, the Tn903.1 fragment is more efficient than the Tn3.1 fragment at maintaining cells with plasmid, because kanamycin persists in the culture media, whereas ampicillin is rapidly degraded (Cumming et al. 2022; Korpimäki et al. 2003). Finally, increased plasmid maintenance did not result in higher production titers.

### Maintenance of pET plasmids in C41(DE3)

**Fig. 2:**
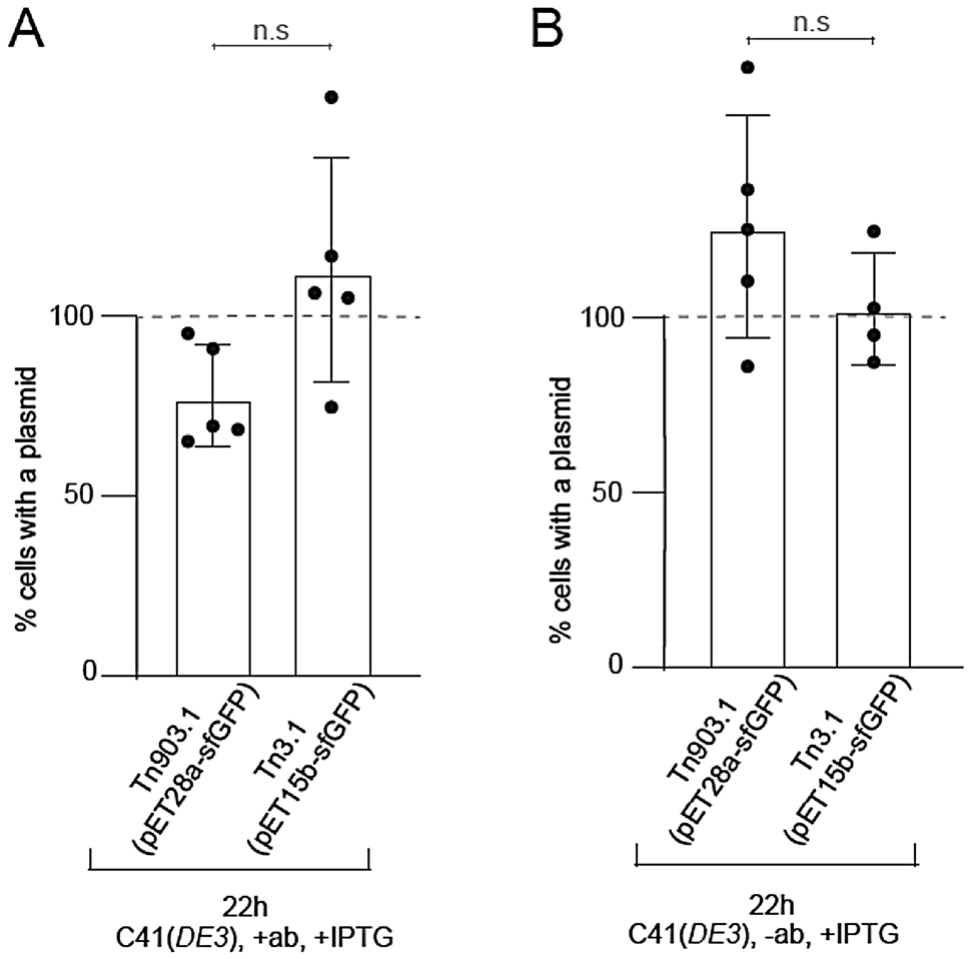
Maintenance of pET plasmids in C41(*DE3*) harboring Tn903.1 and Tn3.1 (cultivated in LB media with and without antibiotics). **A** Plasmid maintenance for C41(*DE3*) harboring either pET28a-sfGFP (Tn903.1) or pET15b-sfGFP (Tn3.1), with antibiotics and induction. Plasmid maintenance was determined from liquid cultures grown without antibiotics, then plated on LB agar plates with and without antibiotic selection. Plasmid maintenance = [# colonies on plate with antibiotic / # colonies on plate without antibiotic] × 100. **B** As for panel (A) except antibiotics were omitted. A statistically significant difference of *p* < 0.05 (two-tailed Student’s *t*-test) is denoted by *. n.s means that the difference was not statistically significant

A large body of work has suggested that the burden of recombinant protein production contributes to poor plasmid maintenance (Bentley et al. 1990; Dumon-Seignovert et al. 2004; Pan and Malcolm 2000; Popov et al. 2009; Silva et al. 2012). We too observed this when we induced sfGFP and Mth1 in BL21(*DE3*) for + 20 h (both with and without antibiotics) (see above). We investigated whether we could increase the efficiency of plasmid maintenance in these induction conditions by reducing the burden of recombinant protein production. In this set of experiments, we switched to the C41(*DE3*) strain (Miroux and Walker 1996), which has lowered levels of the T7 RNA polymerase and a lower protein production rate (Kwon et al. 2015). pET28a-sfGFP (Tn903.1) and pET15b-sfGFP (Tn3.1) were transformed into C41(*DE3*), and sfGFP production was again induced with 0.5 mM IPTG for 20 h (22 h of cultivation). Fluorescence readings indicated that the expression levels of sfGFP in C41(*DE3*) were > 4 times lower than that those observed in BL21(*DE3*) (Supplementary information Figure S5). Both pET28a-sfGFP (Tn903.1) and pET15b-sfGFP (Tn3.1) were efficiently maintained with approximately 100% efficiency in the C41(*DE3*) strain during recombinant protein production experiments when antibiotics were present in the culture media (Fig. 2A). We repeated the above experiment in the absence of antibiotics in the culture media and were surprised to observe that pET28a-sfGFP (Tn903.1) and pET15b-sfGFP (Tn3.1) were still maintained with approximately 100% efficiency in C41(*DE3*) over a 20-h induction (Fig. 2B). Taken together, these data indicate that over long inductions, the C41(*DE3*) strain maintains pET plasmids efficiently, irrespective of the resistance fragment used (Tn903.1 and Tn3.1), or the presence or absence of antibiotics.

## Discussion

The Tn3.1-based and Tn903.1-based fragments are essential components of pET expression plasmids. They confer resistance to antibiotics and enable selection of bacterial cells with a plasmid following transformation (by plating on the appropriate antibiotic). They also enable maintenance of the plasmid during long cultivations (by including the appropriate antibiotic in the growth media). In this study, we have investigated how efficiently pET plasmids are maintained when using these fragments.

Initially, we used the most common pET resources and protocols, such as the pET28a and pET15b expression plasmids, the BL21(*DE3*) strain, and standard media/induction protocols (Aguilar Lucero et al. 2021; Novagen 2005; Rosano et al. 2019; Shilling et al. 2020; Structural Genomics Consortium et al. 2008). We then monitored plasmid maintenance by plating the cells with and without antibiotics. This allowed us to determine the percentage of cells in the culture that were resistant to a given antibiotic (a proxy for plasmid maintenance). The data indicated that, prior to induction of recombinant protein production, most cells contained a pET plasmid for long cultivation times (i.e., 22 h). This observation was irrespective of whether or not antibiotics were present in the culture media (a phenomenon known as the plasmid paradox (Carroll and Wong 2018)). However, when recombinant protein production was induced, most BL21(*DE3*) cells contained a pET plasmid for short cultivation times (i.e., 2 h post induction) but not over longer cultivation times (i.e., 20 h post induction). In these conditions, we observed that pET expression plasmids containing Tn903.1 (and selected with kanamycin) were more efficiently maintained in BL21(*DE3*) than those containing Tn3.1 (and selected with ampicillin).

**Fig. 3:**
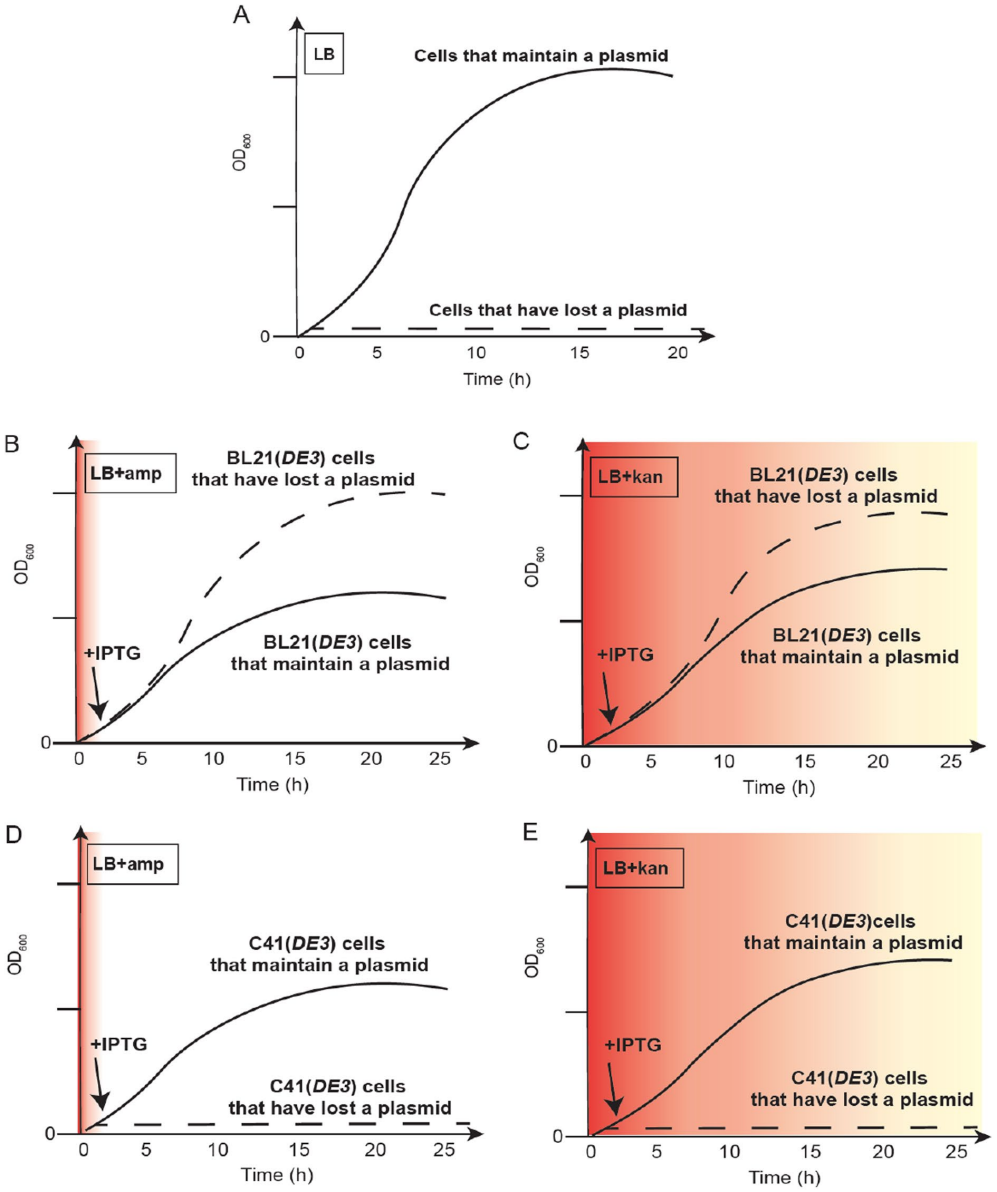
An explanation for how cells that have lost the plasmid contribute to the biomass. **A** In the absence of recombinant protein production, cells that have lost a plasmid following random segregation do not have a detectable fitness advantage compared to those that have a plasmid and do not contribute to the biomass. **B** When BL21(*DE3*) cells are induced to produce a recombinant protein from a pET plasmid containing Tn3.1 (and selected on ampicillin), the biomass contains a large proportion of cells that have lost a plasmid. Cells that have lost a plasmid have a detectable fitness advantage, compared to those that have a plasmid and are not selected against because ampicillin is short-lived in the culture media (depicted by red zone). **C** When BL21(*DE3*) cells are induced to produce a recombinant protein from a pET plasmid containing Tn903.1 (and selected on kanamycin), the biomass still contains a proportion of cells that have lost a plasmid even though kanamycin is long-lived in the culture media (depicted by red zone). The reason why this occurs is not known but may relate to community protection. **D** When C41(*DE3*) cells are induced to produce a recombinant protein from a pET plasmid containing Tn3.1 (and selected on ampicillin), cells that have lost a plasmid do not accumulate as they do not have a detectable fitness advantage compared to those that have a plasmid. **E** When C41(*DE3*) cells are induced to produce a recombinant protein from a pET plasmid containing Tn903.1 (and selected on kanamycin), cells that have lost a plasmid do not accumulate as they do not have a detectable fitness advantage compared to those that have a plasmid

Why are plasmids containing Tn903.1 (and selected with kanamycin) more efficiently maintained in BL21(*DE3*) than those containing Tn3.1 (and selected with ampicillin)? Decades old literature has indicated that pBR322-based plasmids (which the pET plasmids are derived from) are randomly segregated to daughter cells during cell division (Chiang and Bremer 1988). As the principle of random segregation also implies, daughters can receive unequal amounts of the plasmid pool at each division. Chiang and Bremer estimated that 1:10,000 divisions would yield a cell without a plasmid (Chiang and Bremer 1988). Thus, over the course of a 24-h incubation, plasmid-less cells would accumulate and could represent a small proportion of the total population (Fig. 3A). However, in the context of a protein production experiment, the plasmid-less cells would be freed from the metabolic burdens of both plasmid replication and plasmid-related protein production, and have a significant growth advantage. Plasmid-less cells would eventually outgrow their plasmid-containing counterparts in the population during a long induction. It is therefore important to select against plasmid-less cells during recombinant protein production experiment, by including antibiotics in the growth media. The scenario above stated explains why plasmids containing Tn3.1 (and selected with ampicillin) are less efficiently maintained in BL21(*DE3*) than those containing Tn903.1 (and selected with kanamycin). Ampicillin is short-lived in the culture media while kanamycin is long-lasting. Thus, BL21(*DE3*) cells that lose a plasmid containing Tn3.1 are not selected against and can outgrow those that maintain a plasmid (Fig. 3B). In contrast, BL21(*DE3*) cells that lose a plasmid containing Tn903.1 are selected against. All said, it is worth noting that even though kanamycin was still active in the culture media (i.e., above the MIC_90_ of BL21(*DE3*)), we did not observe 100% plasmid maintenance (Fig. 3C). The reasons why some plasmid-less cells can accumulate in the culture are not fully understood at this point in time. It may relate to the fact that a population of cells can survive in the spent culture media through community protection.

pET expression plasmids are typically chosen based on salient features, such as purification, detection or secretion tags rather than on the antibiotic resistance cassette. One notable exception is when producing recombinant membrane or secreted proteins, where preference is given to the Tn903.1 fragment, as the Tn3.1 fragment encodes for the periplasmic ß-lactamase that needs the same machinery as the recombinant protein for its biogenesis (Kaderabkova et al. 2022). In contrast, the aminoglycoside-3’-phosphotransferase encoded by Tn903.1 is cytosolic and doesn’t compete with the recombinant protein for the secretion machinery. The data presented here suggest that more consideration should be given to the choice of antibiotic resistance fragment when choosing a pET expression plasmid, as the Tn3.1- and Tn903.1-type fragments perform differently and need to be matched with the production experiment. Specifically, BL21(*DE3*) maintained pET plasmids with a Tn903.1-type fragment more efficiently than those containing a Tn3.1-type fragment when the appropriate antibiotic was present in the culture media. Even though better plasmid maintenance was observed when using a Tn903.1-type fragment, we did not observe increased production titers. This can be attributed to the fact that, in BL21(*DE3*), the T7 system is either down-regulated or shut-off by mutations over long induction times (James et al. 2021; Kwon et al. 2015; Schlegel et al. 2015).

If poor plasmid maintenance is not acceptable, one solution identified in this study is to switch strain background. When we used C41 (*DE3*), we observed that the plasmid maintenance was unaffected by induction of recombinant protein production, culturing times, presence or absence of antibiotics, and resistance fragments chosen (Fig. 3D and E). The reason why C41(*DE3*) was able to better maintain plasmids compared to BL21(*DE3*) is because it has lower levels of the T7 RNA polymerase, and thus recombinant protein production places a lower metabolic burden on the cell. As a consequence, plasmid-less C41(*DE3*) do not outgrow the plasmid-containing cells during long inductions. One caveat to consider, is that it may be possible to place a metabolic load on C41(*DE3*) and enable plasmid-less cells to take over the culture, if the recombinant protein is particularly toxic to the cell, or if the pET plasmid lacks genetic modules that help regulate inducible expression (Dumon-Seignovert et al. 2004). Efficient plasmid maintenance does come at a cost, as we observed that titers of recombinant sfGFP were > 4 times lower in C41(*DE3*) than they were in BL21(*DE3*) after 20 h of induction. These observations highlight the conundrum. It is not possible to choose a strain which efficiently maintains the expression plasmid and produces high titers.

A large body of work has investigated factors that contribute to plasmid maintenance. This body of work has been carried out using a variety of different plasmids, cell strains, recombinant proteins, antibiotic resistance fragments and culturing conditions. The knowledge is not immediately transferable to the pET system, which is the gold standard for recombinant protein production (Heyde and Nørholm 2021; Rosano et al. 2019; Rosano and Ceccarelli 2014; Shilling et al. 2020). In this study, we have addressed this knowledge gap by investigating how efficiently pET plasmids are maintained using the Tn3.1-type and Tn903.1-type fragments. The study identified factors that contribute to both efficient and poor maintenance. Based on this work, we have two simple tips for efficiently maintaining pET expression plasmids:

- #1: Use a strain with lowered levels of the T7 RNA polymerase, such as C41(*DE3*). pET plasmids will be efficiently maintained over long induction times with both the Tn3.1 and Tn903.1 genetic fragments, regardless of whether antibiotics are present during cultivation. This strategy will most likely lower the overall titers of recombinant protein but it will also allow you to work in the absence of antibiotic selection while ensuring high levels of plasmid maintenance. This is particularly relevant to industrial fermentations where antibiotic residues and metabolites are problematic.
- Tip #2: If a strain with higher levels of T7 RNA polymerase strain is necessary, such as BL21(*DE3*)), keep induction times short or use a plasmid containing a Tn903.1-type fragment and select with kanamycin. Keeping inductions short will also help to avoid mutations that either down-regulate or shut-off the T7 RNA polymerase (James et al. 2021; Kwon et al. 2015a; Schlegel et al. 2015).

### Supplementary Information

Below is the link to the electronic supplementary material.

Supplementary file1 (DOCX 684 KB)

## Data Availability

All primary data and materials are available from the authors upon request.

## Abbreviations

LB: Lysogeny broth
sfGFP: Superfolder green fluorescent protein
OD_600_: Optical density at 600 nm
PCR: Polymerase chain reaction
IPTG: Isopropyl-ß-D-1-thiogalactopyranoside

## References

[CR1] Aguilar Lucero, D., Cantoia, A., Ceccarelli, E.A., Rosano, G.L., 2021. Starting a new recombinant protein production project in Escherichia coli, in: Methods in Enzymology. Elsevier, pp. 3–18. 10.1016/bs.mie.2021.08.019 34752291

[CR2] Baheri HR, Hill GA, Roesler WJ (2001). Modelling plasmid instability in batch and continuous fermentors. Biochem Eng J.

[CR3] Bentley WE, Mirjalili N, Andersen DC, Davis RH, Kompala DS (1990). Plasmid-encoded protein: The principal factor in the “metabolic burden” associated with recombinant bacteria. Biotechnol Bioeng.

[CR4] Bolivar F, Rodriguez RL, Betlach MC, Boyer HW (1977). Construction and characterization of new cloning vehicles. I. Ampicillin-resistant derivatives of the plasmid pMB9. Gene.

[CR5] Carroll AC, Wong A (2018). Plasmid persistence: costs, benefits, and the plasmid paradox. Can J Microbiol.

[CR6] Chamberlin M, Ring J (1973). Characterization of T7-specific ribonucleic acid polymerase. 1. General properties of the enzymatic reaction and the template specificity of the enzyme. J Biol Chem.

[CR7] Chiang C-S, Bremer H (1988). Stability of pBR322-derived plasmids. Plasmid.

[CR8] Cumming AJ, Khananisho D, Harris R, Bayer CN, Nørholm MHH, Jamshidi S, Ilag LL, Daley DO (2022). Antibiotic-efficient genetic cassette for the TEM-1 β-lactamase that improves plasmid performance. ACS Synth Biol.

[CR9] Daley DO, Rapp M, Granseth E, Melén K, Drew D, von Heijne G (2005). Global topology analysis of the Escherichia coli inner membrane proteome. Science.

[CR10] Dumon-Seignovert L, Cariot G, Vuillard L (2004). The toxicity of recombinant proteins in Escherichia coli: a comparison of overexpression in BL21(DE3), C41(DE3), and C43(DE3). Protein Expr Purif.

[CR11] Gad H, Koolmeister T, Jemth A-S, Eshtad S, Jacques SA, Ström CE, Svensson LM, Schultz N, Lundbäck T, Einarsdottir BO, Saleh A, Göktürk C, Baranczewski P, Svensson R, Berntsson RP-A, Gustafsson R, Strömberg K, Sanjiv K, Jacques-Cordonnier M-C, Desroses M, Gustavsson A-L, Olofsson R, Johansson F, Homan EJ, Loseva O, Bräutigam L, Johansson L, Höglund A, Hagenkort A, Pham T, Altun M, Gaugaz FZ, Vikingsson S, Evers B, Henriksson M, Vallin KSA, Wallner OA, Hammarström LGJ, Wiita E, Almlöf I, Kalderén C, Axelsson H, Djureinovic T, Puigvert JC, Häggblad M, Jeppsson F, Martens U, Lundin C, Lundgren B, Granelli I, Jensen AJ, Artursson P, Nilsson JA, Stenmark P, Scobie M, Berglund UW, Helleday T (2014). MTH1 inhibition eradicates cancer by preventing sanitation of the dNTP pool. Nature.

[CR12] Geissmann Q (2013). OpenCFU, a New free and open-source software to count cell colonies and other circular objects. PLoS ONE.

[CR13] Golomb M, Chamberlin M (1974). Characterization of T7-specific ribonucleic acid polymerase. IV. Resolution of the major in vitro transcripts by gel electrophoresis. J Biol Chem.

[CR14] Heyde SAH, Nørholm MHH (2021). Tailoring the evolution of BL21(DE3) uncovers a key role for RNA stability in gene expression toxicity. Commun Biol.

[CR15] James J, Yarnall B, Koranteng A, Gibson J, Rahman T, Doyle DA (2021). Protein over-expression in Escherichia coli triggers adaptation analogous to antimicrobial resistance. Microb Cell Fact.

[CR16] Kaderabkova N, Bharathwaj M, Furniss, RCD, Gonzalez D, Palmer T, Mavridou DAI, (2022) The biogenesis of β-lactamase enzymes. Microbiology 168. 10.1099/mic.0.001217 PMC10235803 35943884

[CR17] Korpimäki T, Kurittu J, Karp M (2003). Surprisingly fast disappearance of beta-lactam selection pressure in cultivation as detected with novel biosensing approaches. J Microbiol Methods.

[CR18] Kwon S-K, Kim SK, Lee D-H, Kim JF (2015). Comparative genomics and experimental evolution of Escherichia coli BL21(DE3) strains reveal the landscape of toxicity escape from membrane protein overproduction. Sci Rep.

[CR19] Mierendorf RC, Morris BB, Hammer B, Novy RE (1998). Expression and purification of recombinant proteins using the pET system. Methods Mol Med.

[CR20] Miroux B, Walker JE (1996). Over-production of proteins in Escherichia coli: mutant hosts that allow synthesis of some membrane proteins and globular proteins at high levels. J Mol Biol.

[CR22] Novagen, 2005. pET System Manual 11th Edition.

[CR23] Oka A, Sugisaki H, Takanami M (1981). Nucleotide sequence of the kanamycin resistance transposon Tn903. J Mol Biol.

[CR24] Pan S, Malcolm BA (2000). Reduced background expression and improved plasmid stability with pET vectors in BL21 (DE3). Biotechniques.

[CR25] Popov M, Petrov S, Kirilov K, Nacheva G, Ivanov I (2009). Segregational Instability in *E. Coli* of expression plasmids carrying human interferon gamma gene and its 3’-End truncated variants. Biotechnol Biotechnol Equip.

[CR26] Rosano GL, Morales ES, Ceccarelli EA (2019). New tools for recombinant protein production in Escherichia coli: A 5-year update. Protein Sci.

[CR27] Rosano GL, Ceccarelli EA, (2014) Recombinant protein expression in Escherichia coli: advances and challenges. Front Microbiol 5. 10.3389/fmicb.2014.00172 PMC4029002 24860555

[CR28] Rosenberg AH, Lade BN, Dao-shan C, Lin S-W, Dunn JJ, Studier FW (1987). Vectors for selective expression of cloned DNAs by T7 RNA polymerase. Gene.

[CR29] Schlegel S, Genevaux P, de Gier J-W (2015). De-convoluting the genetic adaptations of E. coli C41(DE3) in real time reveals how alleviating protein production stress improves yields. Cell Rep.

[CR30] Shilling PJ, Mirzadeh K, Cumming AJ, Widesheim M, Köck Z, Daley DO (2020). Improved designs for pET expression plasmids increase protein production yield in Escherichia coli. Communications Biology.

[CR31] Sieben M, Steinhorn G, Müller C, Fuchs S, Ann Chin L, Regestein L, Büchs J (2016). Testing plasmid stability of *Escherichia coli* using the Continuously Operated Shaken BIOreactor System. Biotechnol Prog.

[CR32] Silva F, Queiroz JA, Domingues FC (2012). Evaluating metabolic stress and plasmid stability in plasmid DNA production by Escherichia coli. Biotechnol Adv.

[CR33] Structural Genomics Consortium, Architecture et Fonction des Macromolécules Biologiques, Berkeley Structural Genomics Center, China Structural Genomics Consortium, Integrated Center for Structure and Function Innovation, Israel Structural Proteomics Center, Joint Center for Structural Genomics, Midwest Center for Structural Genomics, New York Structural GenomiX Research Center for Structural Genomics, Northeast Structural Genomics Consortium, Oxford Protein Production Facility, Protein Sample Production Facility, Max Delbrück Center for Molecular Medicine, RIKEN Structural Genomics/Proteomics Initiative, SPINE2-Complexes, 2008. Protein production and purification. Nat Methods 5, 135–146

[CR34] Studier WF, Rosenberg, AH, Dunn JJ, Dubendorff JW, 1990. Use of T7 RNA polymerase to direct expression of cloned genes, in: Methods in Enzymology. Elsevier, pp. 60–89. 10.1016/0076-6879(90)85008-c 2199796

[CR35] Studier FW, Moffatt BA (1986). Use of bacteriophage T7 RNA polymerase to direct selective high-level expression of cloned genes. J Mol Biol.

[CR36] Sutcliffe JG (1979). Complete nucleotide sequence of the Escherichia coli plasmid pBR322. Cold Spring Harb Symp Quant Biol.

[CR37] Umezawa H (1979). Studies on aminoglycoside antibiotics: enzymic mechanism of resistance and genetics. Jpn J Antibiot.

[CR38] Watson JF, García-Nafría J (2019). In vivo DNA assembly using common laboratory bacteria: A re-emerging tool to simplify molecular cloning. J Biol Chem.

