# Supplementary material for: Tips for efficiently maintaining pET expression plasmids
Source: Curr Genet. 2023 Nov 8;69(4-6):277–87. doi: 10.1007/s00294-023-01276-0 (PMC10716060; doi:10.1007/s00294-023-01276-0)
Supplement: Supplementary file 1 — Supplementary file1 (DOCX 684 KB) [file 294_2023_1276_MOESM1_ESM.docx]

Supplementary information

**Tips for efficiently maintaining pET expression plasmids**

Diana Khananisho^1,#^, Alister J. Cumming^1,#^, Daria Kulakova^1^,

Patrick J. Shilling^1^ and Daniel O. Daley^1^

^1^ Department of Biochemistry and Biophysics, Stockholm University, Sweden.

**Table S1. A list of primers used in the study.**

| Primer | Sequence |
| --- | --- |
| Amplification of Tn903.1 for insertion into pET15b | 5’- GCTGTCAAACATGAGAACATGAACAATAAAACTGTCTGC - 3’  5’- CCTTTGATCTTTTCTACCTTAGAAAAACTCATCGAGCATC-3' |
| Amplification of Tn3.1 for insertion into pET28a | 5’- CGTTAAGGGATTTTGGTTTCTTGAAGACGAAAGGGCCTCGT - 3’  5’- CCGCTCATGAATTAATTGGGGTCTGACGCTCAGTGG -3' |
| Amplification of pET28a plasmid fragment without Tn903.1 | 5’- ACCAAAATCCCTTAACGTGAGTTTTC - 3’  5’- AATTAATTCATGAGCGGATACATATTTG -3' |
| Sequencing primers for Tn3.1 in pET28a | 5’- CGGCCTATTGGTTAAAAAATG - 3’ (reverse)  5’- GTTGGTAGCTCTTGATCCG -3' (forward) |
| Amplification of pET15b plasmid fragment without Tn3.1 | 5’- TTCTCATGTTTGACAGCTTATCATCG - 3’  5’- GTAGAAAAGATCAAAGGATCTTCTTG -3' |
| Sequencing primers for Tn903.1 in pET15b | 5’- GGTATCTGCGCTCTGC- 3’ (reverse)  5’- CCGGCATAACCAAGCC -3' (forward) |

**
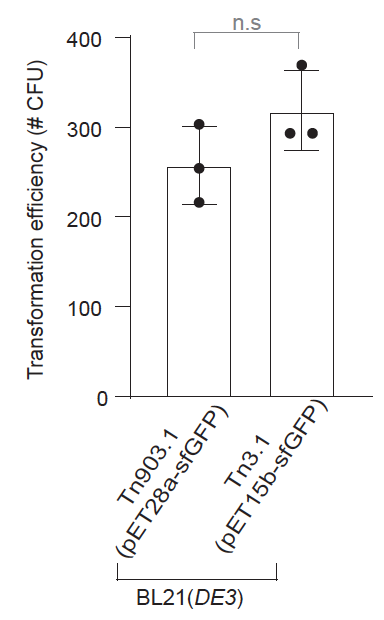
**

**Figure S1. Transformation efficiency of pET expression plasmids with either the Tn903.1 or Tn3.1 fragments.**

Transformation efficiency was determined by transforming 50 ng of each pET expression plasmid in BL21(*DE3*) and counting colonies on LB-agar containing 50 μg/mL of kanamycin for pET28a-sfGFP (Tn903.1) or 100 ampicillin μg/mL for pET15b-sfGFP (Tn3.1). Data presented as mean ± s.d. (n ≽ 3). A statistically significant difference of p < 0.05 (two-tailed Student’s t-test) is denoted by *. n.s means that the difference was not statistically significant.

**
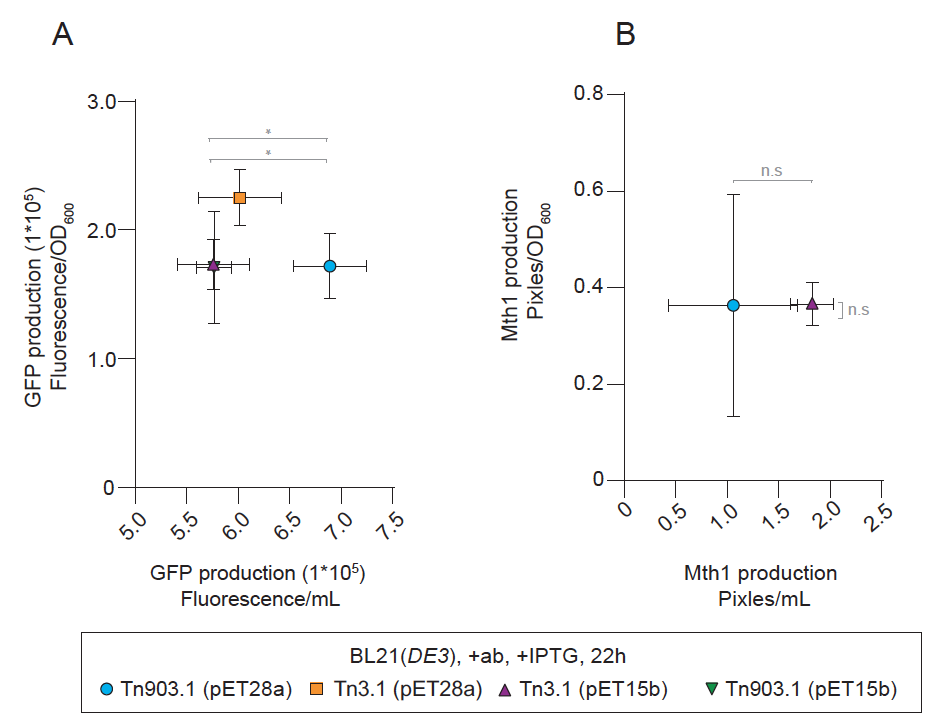
**

**Figure S2. Protein production titres using pET expression plasmids with either the Tn903.1 or Tn3.1 fragment.**

**(A)** sfGFP production was quantified by measuring whole cell fluorescence from 1 mL of culture (x-axis) or normalizing it by the OD_600_ of the culture (y-axis). These experiments were carried out over a 22-hour time period in BL21(*DE3*) harbouring pET28a-sfGFP (Tn903.1 or Tn3.1) and pET15b-sfGFP (Tn3.1 or Tn903.1). The data indicate that there were no major differences in protein yields between BL21(*DE3*) cells harbouring pET expression plasmids with either the Tn903.1 fragment or the Tn3.1 fragment. (**B)** Mth1 production was quantified by Western blotting whole cells from 1 mL of culture (x-axis) or normalizing the signal by the OD_600_ of the culture (y-axis). These experiments were carried out over a 22-hour time period in BL21(*DE3*) harbouring pET28a-Mth1 (Tn903.1) and pET15b-Mth1 (Tn3.1). The data indicate that there is no obvious correlation between plasmid maintenance and protein production titres. Data presented as mean ± s.d. (n ≽ 3). A statistically significant difference of p < 0.05 (two-tailed Student’s t-test) is denoted by *. n.s means that the difference was not statistically significant. Note that similar experiments with Tn3.1 in panel B were presented in (Cumming et al., 2022). They were repeated here for reference.


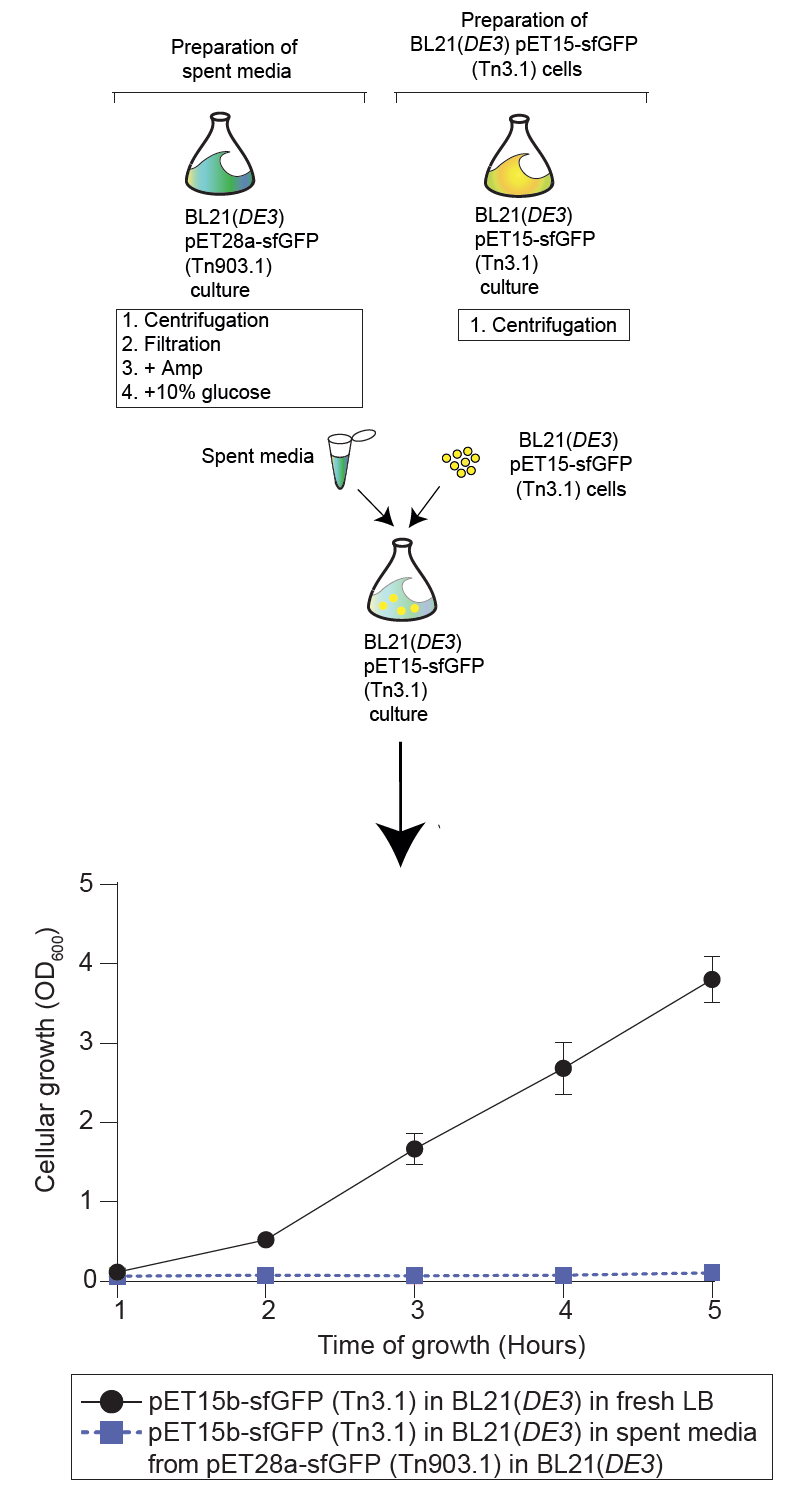


**Figure S3. Kanamycin is active in the culture media over long cultivation times**

Schematic representation of the experimental procedure used to determine whether kanamycin persists in the culture media. BL21(*DE3*) cells harbouring pET28a-sfGFP (Tn903.1) were inoculated in LB with 50 μg /mL kanamycin and grown overnight. The ‘spent media’ was prepared by removing cells by centrifugation and filtration, then supplementing it with 10% (w/v) glucose and 100 μg /mL ampicillin to ensure that any remnant cells harbouring pET28a-sfGFP (Tn903.1) were not viable. To determine if kanamycin was still active in the spent media, BL21(*DE3*) cells harbouring pET15b-sfGFP (Tn3.1) were inoculated into the spent media, and growth was monitored by taking OD_600_ measurements were taken every hour for five hours. The OD_600_ of BL21(*DE3*) cells harbouring pET15b-sfGFP (Tn3.1) in spent media was plotted against time. The experiment revealed that the cells harbouring pET15b-sfGFP (Tn3.1) could not grow in the spent media from BL21(*DE3*) cells harbouring pET28a-sfGFP (Tn903.1). Thus, kanamycin is still active after 20+ hours of culturing with plasmids containing Tn903.1. As a control for cell viability, BL21(*DE3*) cells harbouring pET15b-sfGFP (Tn3.1) did grow in fresh LB media containing 100 μg /mL ampicillin. Note that the MIC_90_ of BL21(DE3) is 10 μg/mL (Supplementary information Figure S6).

**
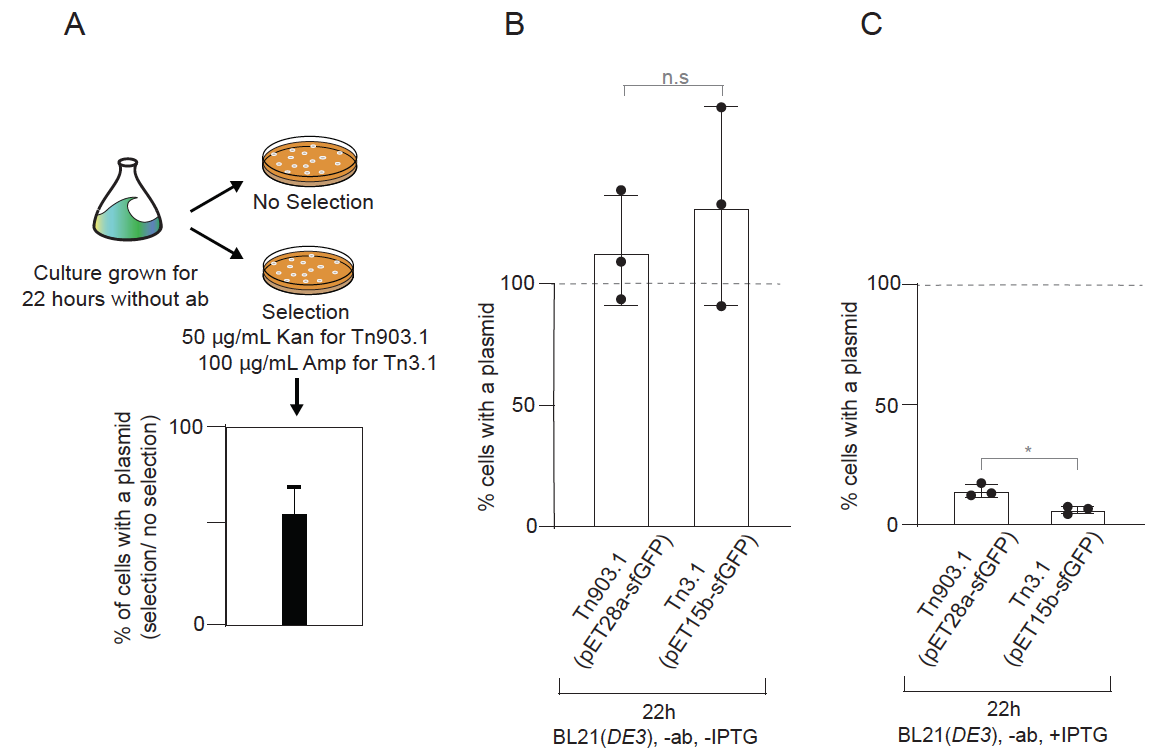
**

**Figure S4 Maintenance of pET plasmids in BL21(*DE3*) harbouring Tn903.1 and Tn3.1 (cultivated in LB media lacking antibiotics).**

**(A)** Schematic representation of the experimental workflow (adapted from (Cumming et al., 2022). Plasmid maintenance was determined from liquid cultures grown in the absence of antibiotics, then plated on LB agar plates with and without antibiotic selection. Plasmid maintenance = [# colonies on plate with antibiotic / # colonies on plate without antibiotic] x100. **(B)** Plasmid maintenance for BL21(*DE3*) harbouring either pET28a-sfGFP (Tn903.1) or pET15b-sfGFP (Tn3.1) in the absence of induction with IPTG and absence of antibiotic selection. **(C)** As for panel (B) except that cells were induced with 0.5 mM IPTG. Data presented as mean ± s.d. (n ≽ 3). A statistically significant difference of p < 0.05 (two-tailed Student’s t-test) is denoted by *. n.s means that the difference was not statistically significant. Note that similar experiments with Tn3.1 in panels B and C were presented in (Cumming et al., 2022). They were repeated here for reference.


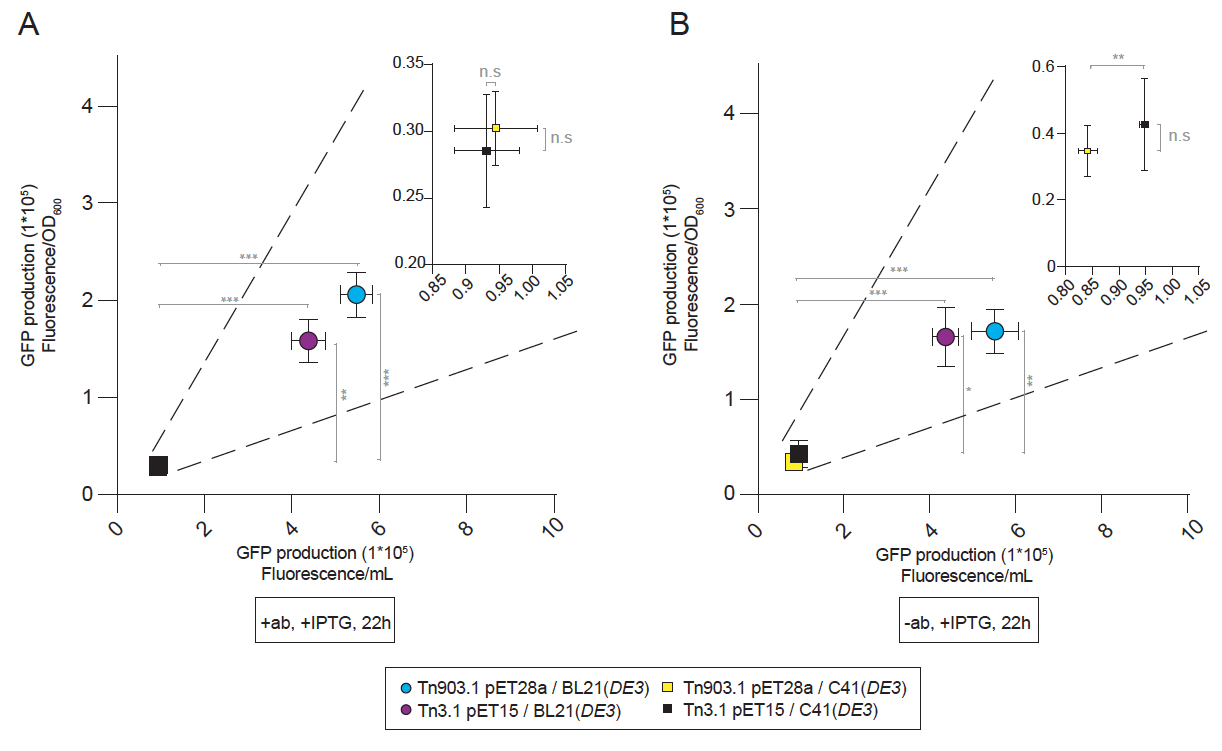


**Figure S5. Protein production titres in different strain backgrounds**

**(A)** sfGFP production was quantified by measuring whole cell fluorescence from 1 mL of culture (x-axis) and normalizing it by the OD_600_ of the culture (y-axis). These experiments were carried out over a 22h time period in BL21(*DE3*) or C41(*DE3*) harbouring pET28a-sfGFP (Tn903.1) or pET15b-sfGFP (Tn3.1) in the presence of antibiotics in the culture media. The data indicate that there were differences in protein yields between BL21(*DE3*) and C41(*DE3*) cells. (**B)** As in (A), except cells were cultured in the absence of selection throughout the experiment. Data presented as mean ± s.d. (n ≽ 3). A statistically significant difference of p < 0.05, p < 0.005 or p < 0.0005 (two-tailed Student’s t-test) is denoted by *, ** and *** respectively. n.s means that the difference was not statistically significant.


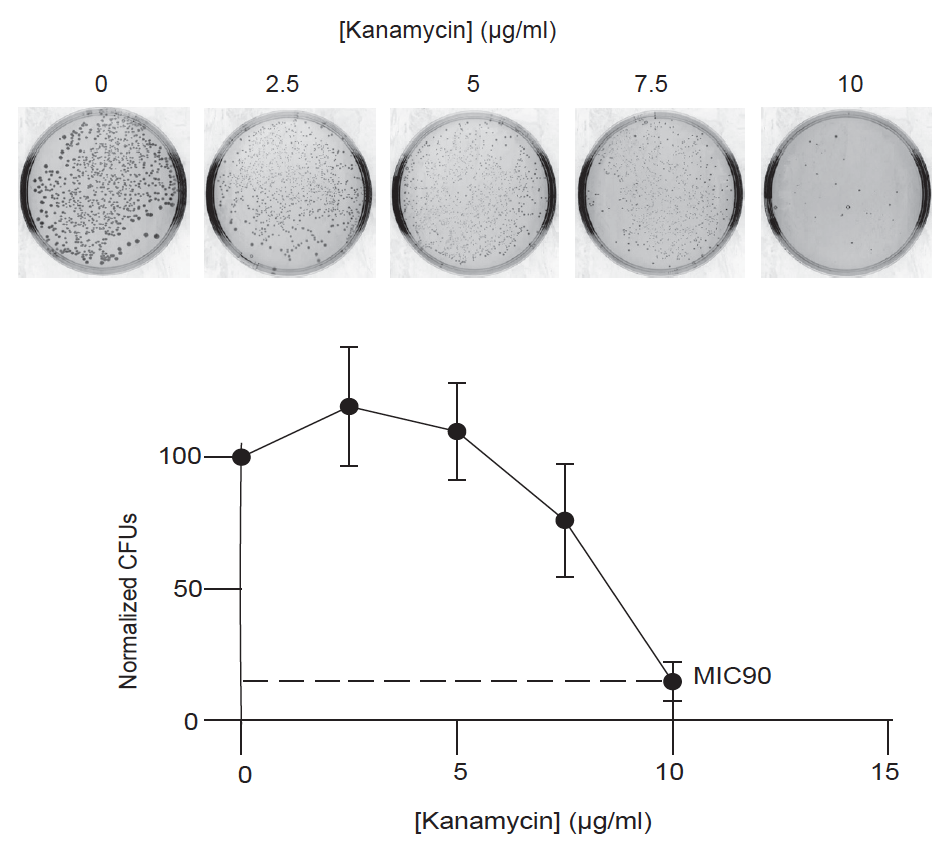


**Figure S6. Calculation of the minimum inhibitor concentration of kanamycin required to kill BL21(*DE3*).**

Top panel, BL21(*DE3*) were plated on LB agar containing increasing concentrations of kanamycin. Bottom panel, colony numbers from were normalised by the number of colonies that grew in the absence of antibiotic. The Minimum Inhibitory Concentrations (MIC_90_’s) required to kill 90 % of cells was extrapolated from the curve (dotted line) and estimated to be 10 μg /mL.

**References**

Cumming, A.J., Khananisho, D., Harris, R., Bayer, C.N., Nørholm, M.H.H., Jamshidi, S., Ilag, L.L., Daley, D.O., 2022. Antibiotic-Efficient Genetic Cassette for the TEM-1 β-Lactamase That Improves Plasmid Performance. ACS Synth Biol 11, 241–253.
